# Inhibition of Autophagy Contributes to Ischemic Postconditioning-Induced Neuroprotection against Focal Cerebral Ischemia in Rats

**DOI:** 10.1371/journal.pone.0046092

**Published:** 2012-09-28

**Authors:** Li Gao, Teng Jiang, Jun Guo, Yi Liu, Guiyun Cui, Lize Gu, Lingying Su, Yingdong Zhang

**Affiliations:** 1 Department of Neurology, Affiliated Nanjing First Hospital, Nanjing Medical University, Nanjing, Jiangsu Province, P. R. China; 2 Department of Biochemistry and Molecular Biology, Nanjing Medical University, Nanjing, Jiangsu Province, P. R. China; 3 Department of Neurology, Affiliated Nanjing Brain Hospital, Nanjing Medical University, Nanjing, Jiangsu Province, P. R. China; 4 Department of Neurology, Affiliated Hospital of Xuzhou Medical College, Xuzhou, Jiangsu Province, P. R. China; Julius-Maximilians-Universität Würzburg, Germany

## Abstract

**Background:**

Ischemic postconditioning (IPOC), or relief of ischemia in a stuttered manner, has emerged as an innovative treatment strategy to reduce programmed cell death, attenuate ischemic injuries, and improve neurological outcomes. However, the mechanisms involved have not been completely elucidated. Recent studies indicate that autophagy is a type of programmed cell death that plays elusive roles in controlling neuronal damage and metabolic homeostasis. This study aims to determine the role of autophagy in IPOC-induced neuroprotection against focal cerebral ischemia in rats.

**Methodology/Principal Findings:**

A focal cerebral ischemic model with permanent middle cerebral artery (MCA) occlusion plus transient common carotid artery (CCA) occlusion was established. The autophagosomes and the expressions of LC3/Beclin 1/p62 were evaluated for their contribution to the activation of autophagy. We found that autophagy was markedly induced with the upregulation of LC3/Beclin 1 and downregulation of p62 in the penumbra at various time intervals following ischemia. IPOC, performed at the onset of reperfusion, reduced infarct size, mitigated brain edema, inhibited the induction of LC3/Beclin 1 and reversed the reduction of p62 simultaneously. Rapamycin, an inducer of autophagy, partially reversed all the aforementioned effects induced by IPOC. Conversely, autophagy inhibitor 3-methyladenine (3-MA) attenuated the ischemic insults, inhibited the activation of autophagy, and elevated the expression of anti-apoptotic protein Bcl-2, to an extent comparable to IPOC.

**Conclusions/Significance:**

The present study suggests that inhibition of the autophagic pathway plays a key role in IPOC-induced neuroprotection against focal cerebral ischemia. Thus, pharmacological inhibition of autophagy may provide a novel therapeutic strategy for the treatment of stroke.

## Introduction

Stroke is the second leading cause of death and a major cause of disability worldwide [1]. Rapid revascularization of the occluded vessels and timely reperfusion is one of the most effective approaches to salvage cerebral ischemic damage. However, restoration of blood flow during reperfusion phase can evoke ischemia-reperfusion (I/R) injury that is not present at ischemia phase but can be modulated only at reperfusion. Efforts have been made to alter the patterns of reperfusion and to alleviate I/R injury. Ischemic postconditioning (IPOC), first reported by Zhao et al. in 2006, represents a promising strategy to reduce I/R injury and attenuate the lethal ischemic damage after stroke [2]. This protective strategy is induced by a repetitive series of brief interruptions of reperfusion applied at the onset of reperfusion after a prolonged period of ischemia. Furthermore, anesthetics and other pharmacological agents can be used as postconditioning stimuli or triggers to elicit protective effects, which is called anesthetic or pharmacological postconditioning [3], [4]. The neuroprotection elicted by postconditioning has been well demonstrated from different laboratories in different species [5]–[7]. In the research field of myocardial ischemia, the intensive research of IPOC has even led to clinical trials [8], [9]. However, the mechanism underlying the effects has yet to be investigated.

Autophagy is the cellular process that mediates lysosomal degradation of long-lived cytoplasmic proteins, initiated under the conditions of differentiation, starvation, or stress such as oxidative stress, endoplasmic reticulum stress and protein aggregate accumulation [10]–[12]. During autophagy, cytoplasmic components are sequestered into double-membrane vesicles called autophagosomes, then fuse with lysosomes to produce single-membraned autophagolysosomes, and degraded by lysosomal hydrolases. LC3 and Beclin 1 are two pacemakers in the autophagic cascade. LC3, the microtubule-associated protein 1A light chain 3, exists in cytosolic form (LC3-I) and membrane-bound form (LC3-II). The ratio of conversion from LC3-I to LC3-II is closely correlated with the extent of autophagosome formation [13]. Beclin 1, first described in a yeast two-hybrid screen as a Bcl-2-interacting protein, is essential for the recruitment of other autophagic proteins during the expansion of pre-autophagosomal membrane [14], [15]. In addition, the autophagic protein p62/SQSTM1 is selectively incorporated into autophagosomes through direct binding to LC3 and efficiently degraded by autophagy. The level of p62 inversely correlates with autophagic activity [16]. Autophagy activation can maintain cellular homeostasis and survival either by purging the cell of dysfunctional organelles, toxic metabolites and intracellular pathogens, or by generating the intracellular building blocks required to preserve vital functions during nutrient deprivation [10], [17]. Simultaneously, autophagy can also trigger non-apoptotic programmed cell death (autophagic cell death) through excessive self-digestion and degradation of essential cellular constituents [17], [18], which is implicated in various physiological and pathological conditions relevant to neurological diseases.

The autophagy-lysosomal pathway is an evolutionarily conserved process of regulated turnover of cellular constituents [10]. Some earlier studies have reported that autophagy is induced in cerebral ischemia in various animal models, including focal or global cerebral ischemia or hypoxia-ischemia models in rats and mice [19]–[24]. However, whether and how autophagy is involved in the IPOC-induced tolerance to cerebral ischemia has not been shown in those studies. At present, we tested our hypothesis in a focal cerebral ischemic model with permanent MCA occlusion plus transient CCA occlusion, giving a systematical review of potential activators and inhibitors of autophagy, in order to explore the contribution of autophagy to IPOC-induced neuroprotection against cerebral ischemia in rats.

## Results

### Activation of autophagy in focal cerebral ischemia

**Figure 1:**
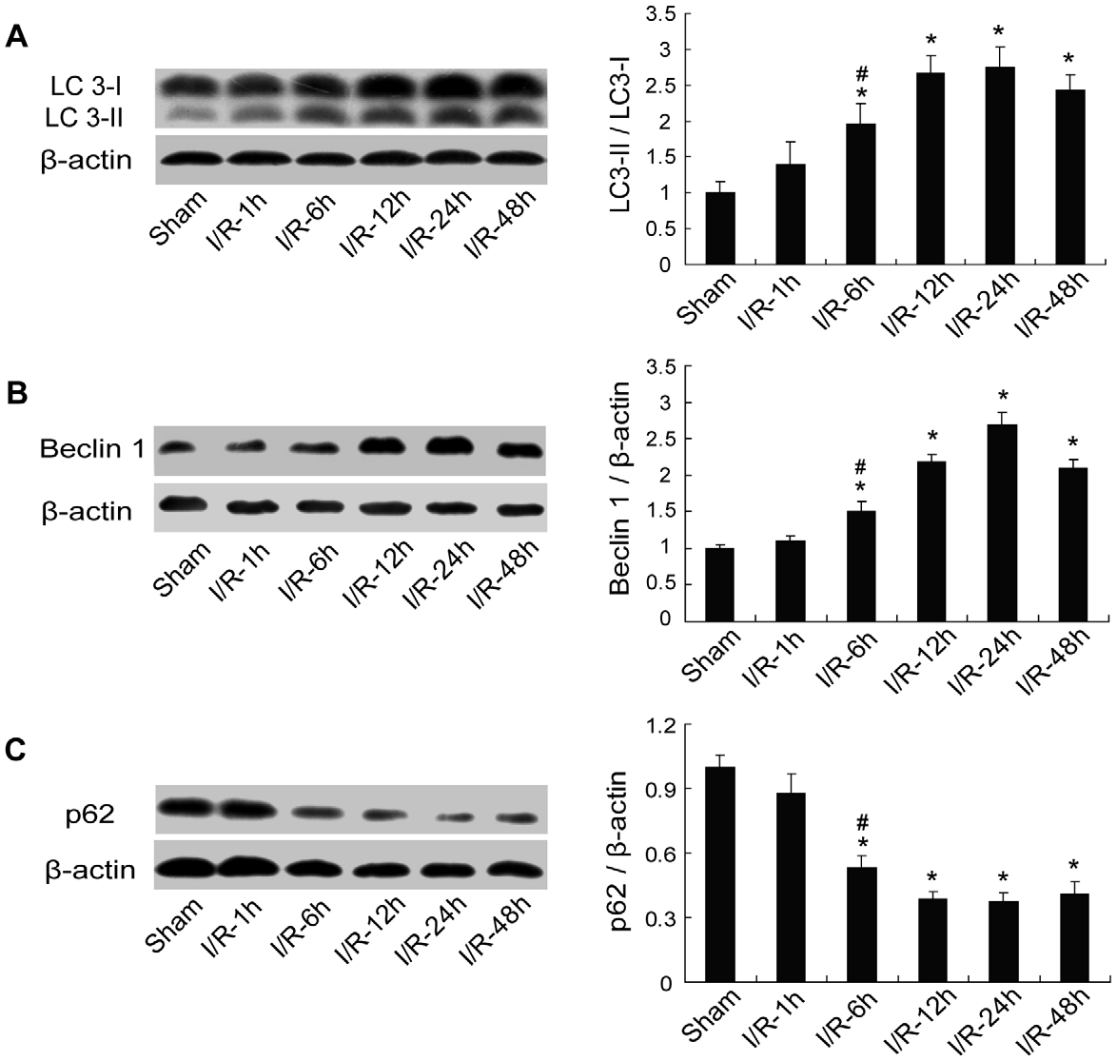
Time-dependent changes of LC3, Beclin 1 and p62 expression in the penumbra following ischemia. Rats were subjected to 30 min ischemia followed by 1, 6, 12, 24, and 48 h reperfusion. Extracts from the sham-operated and ischemic cerebral cortex were separated for immunoblotting. (A–B) Changes of LC3 and Beclin 1 expressions at different times of reperfusion. The ratio of LC3-II/LC3-I and the expression of Beclin 1 increased from 1 h to 48 h with a significant increase starting from 6 h and peaking at 24 h postischemia. (C) Changes of p62 expression at different times of reperfusion. The expression of p62 decreased significantly at 6 h and lasted until 48 h, with a maximum reduction at 24 h postischemia. Levels of β-actin protein were used as the loading control. Bar represents mean ± SEM from 5 rats in each time point. *p<0.05 vs. the Sham group; ^#^ *p*<0.05 vs. I/R-24 h group.

To determine the extent of autophagy activation in the focal cerebral ischemic model, a time course analysis of autophagic markers LC3, Beclin 1, and p62/SQSTM1 was conducted in the penumbra at 1, 6, 12, 24, and 48 h postischemia. The western analysis results revealed the ratio of LC3-II/LC3-I and the expression of Beclin 1 increased as early as 1 h after ischemia, augmented significantly at 6 h, peaked at 24 h and lasted for 48 h postischemia (Figure 1A–B; *p<0.05 vs. the Sham group; ^#^ *p*<0.05 vs. I/R-24 h group). Conversely, the expression of p62 decreased since 1 h and lasted up to 48 h, and there was a significant reduction starting at 6 h and reaching its bottom at 24 h postischemia (Figure 1C; *p<0.05 vs. the Sham group; ^#^ *p*<0.05 vs. I/R-24 h group). These findings suggest an enhancement of autophagy in this focal cerebral ischemic model, and support previous research that autophagy was induced in rats and mice subjected to ischemic insults [19], [20], [23].

### IPOC inhibition of postischemic autophagy

**Figure 2:**
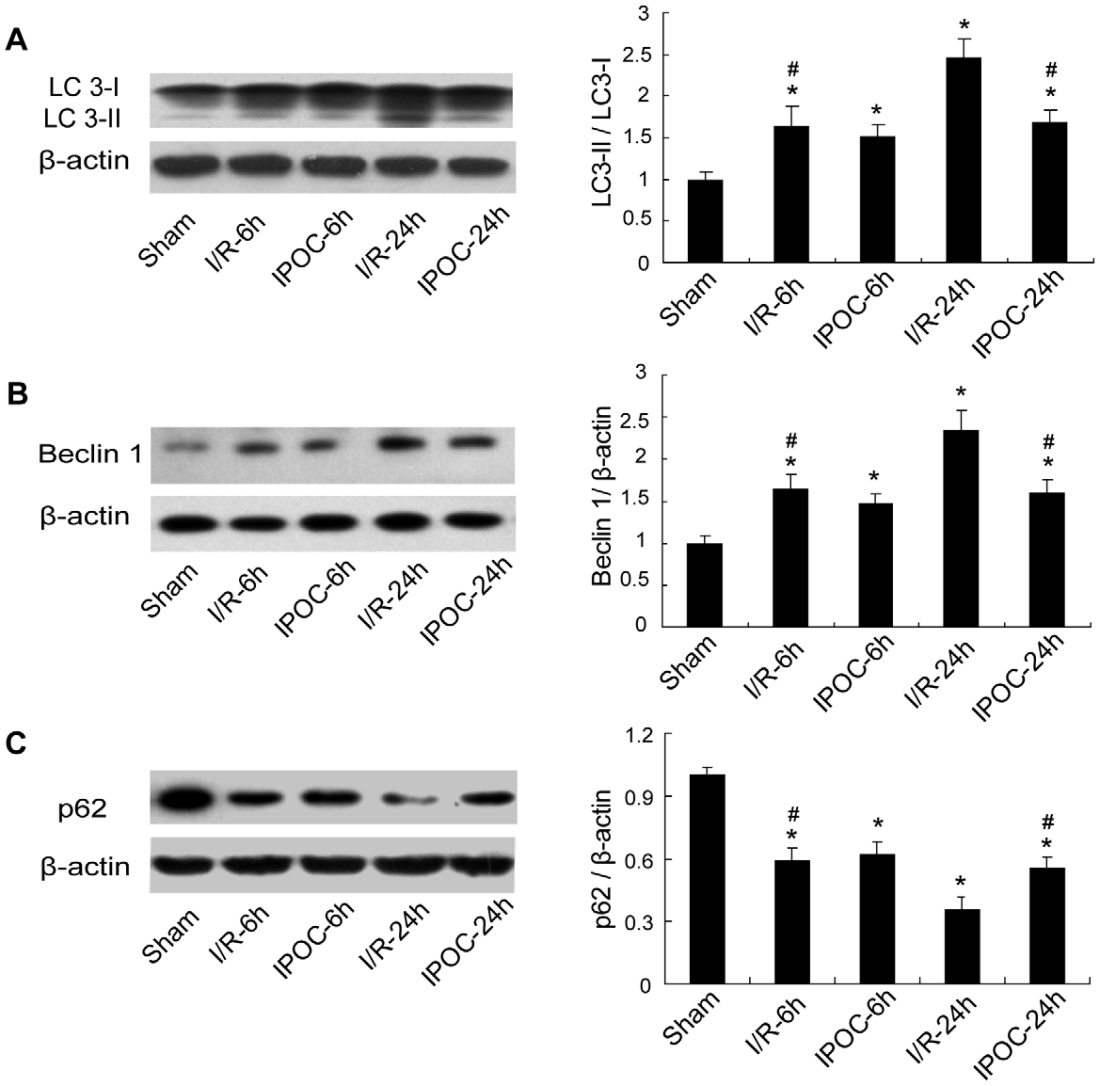
Effects of IPOC on autophagy induction in the penumbra at 6 h and 24 h postischemia. Extracts from the sham-operated and ischemic cerebral cortex in I/R and IPOC groups were separated for immunoblotting at 6 h and 24 h postischemia. (A–C) The expression changes of LC3, Beclin 1 and p62 at 6 h and 24 h after postconditioning treatment. Autophagy is activated in both I/R and IPOC groups, and IPOC eliminated the induction of LC3/Beclin 1 and reversed the reduction of p62 at 24 h but not 6 h postischemia. Levels of β-actin protein were used as the loading control. n = 5 for each group in each time point; *p<0.05 vs. the Sham group; ^#^ *p*<0.05 vs. I/R-24 h group.

As mentioned above, the autophagic activity was upregulated significantly at 6 h and peaked at 24 h after ischemia. Then we examined the expressions of LC3, Beclin 1 and p62 in the Sham, I/R and IPOC groups to evaluate IPOC-induced changes of autophagic activity at these two time-points. As shown in Figure 2A–B, 6 h after ischemia, although the ratio of LC3-II/LC3-I and the protein level of Beclin 1 increased significantly in rats subjected to ischemia with and without postconditioning than that in the sham-operated rats (Figure 2A–B; *p<0.05 vs. the Sham group), there was no significant difference between the two groups. Twenty-four hours after ischemia, the upregulated expressions of LC3 and Beclin 1 were still detected at significant levels in I/R group, but not in rats treated with postconditioning (Figure 2A–B; *p<0.05 vs. the Sham group; ^#^ *p*<0.05 vs. I/R-24 h group). Meanwhile, the expression of p62 decreased greatly in rats subjected to ischemia at 6 h and 24 h, and IPOC significantly blocked the downregulation of p62 only at 24 h postischemia (Figure 2C; *p<0.05 vs. the Sham group; ^#^ *p*<0.05 vs. I/R-24 h group). The immunoblotting results revealed autophagy activation in both I/R and IPOC groups, though varied in extent and persistence. IPOC might attenuate the further induction of autophagy in focal cerebral ischemia.

### Rapamycin partially attenuated IPOC-induced neuroprotection

**Figure 3:**
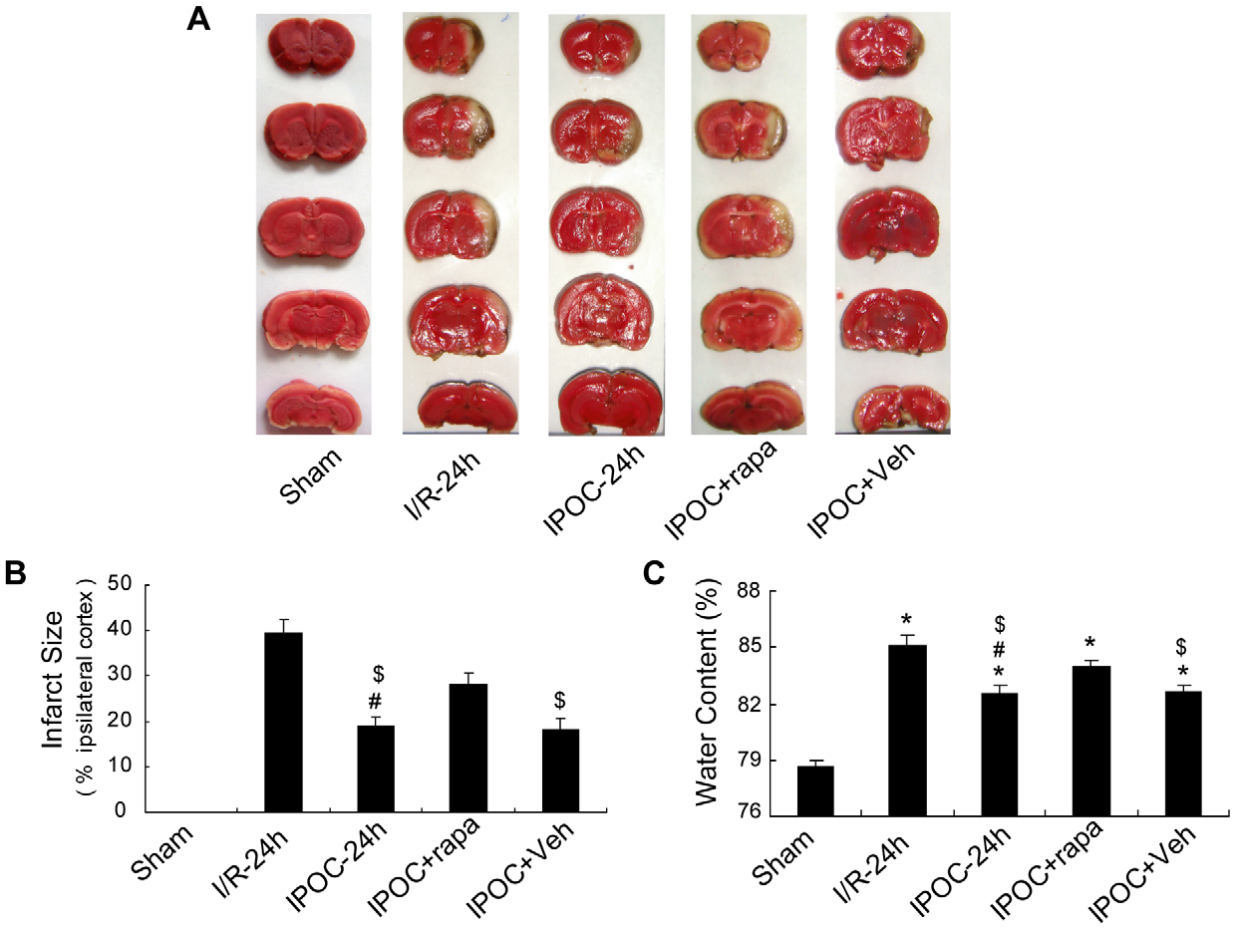
TTC staining and brain edema measurement from rat brains in different groups. Rats were treated with an i.c.v. injection of 35 pmol rapamycin 15 min before postconditioning, and then followed by 24 h reperfusion. (A) Representative infarcts stained with TTC in the Sham, I/R-24 h, IPOC-24 h, IPOC+rapa and IPOC+Veh groups 24 h after stroke. (B) Quantification of infarct size from each group at 24 h after ischemia. (C) Quantification of water content from each group at 24 h after ischemia. IPOC reduced infarct size and mitigated brain edema after stroke, whereas rapamycin partially eliminated the neuroprotection of IPOC. n = 8 for each group. *p<0.05 vs. the Sham group; ^#^ *p*<0.05 vs. I/R-24 h group; ^$^ *p*<0.05 vs. IPOC+rapa group.

To further address the induction of autophagy in IPOC-induced neuroprotection, the autophagy inducer rapamycin was administered before postconditioning. Intracerebral ventricle (i.c.v.) injection of rapamycin at a dose of 35 pmol could effectively induce autophagic activity [25], thus was used in the current study. Considering the controversial role of rapamycin in cerebral ischemia, rapamycin was applied at the onset of reperfusion first to determine its role in this focal cerebral ischemic model. The results of TTC and water edema measurement showed there was no significant difference between the ischemia-only and rapamycin-treated rats at 24 h after ischemia (see Figure S1). The mean infarct size of 39.44±2.86% was detected in the ipsilateral cerebral cortex in ischemia-only rats, while postconditioning significantly reduced the infarct size by approximately 50% at 24 h after ischemia (Figure 3A–B, 36.69±3.05% vs. 19.21±1.84%; ^#^ *p*<0.05 vs. I/R-24 h group), in agreement with previous reports [2], [6]. In addition, measurement of water edema showed that the water content increased to ∼80% in the ischemic brain with postconditioning treatment, but significantly lower than that in the ischemia-only brain (Figure 3C; *p<0.05 vs. the Sham group; ^#^ *p*<0.05 vs. I/R-24 h group), further suggesting that IPOC abrogated ischemia-induced brain injuries. However, these neuroprotective effects were partially reversed by a single i.c.v. injection of rapamycin, resulting in larger infarct size of 28.37±2.30% and more water content as compared with IPOC or IPOC+Veh groups (Figure 3A–C; ^$^ *p*<0.05 vs. IPOC+rapa group). It indicates that rapamycin may attenuate the neuroprotective effects induced by postconditioning.

**Figure 4:**
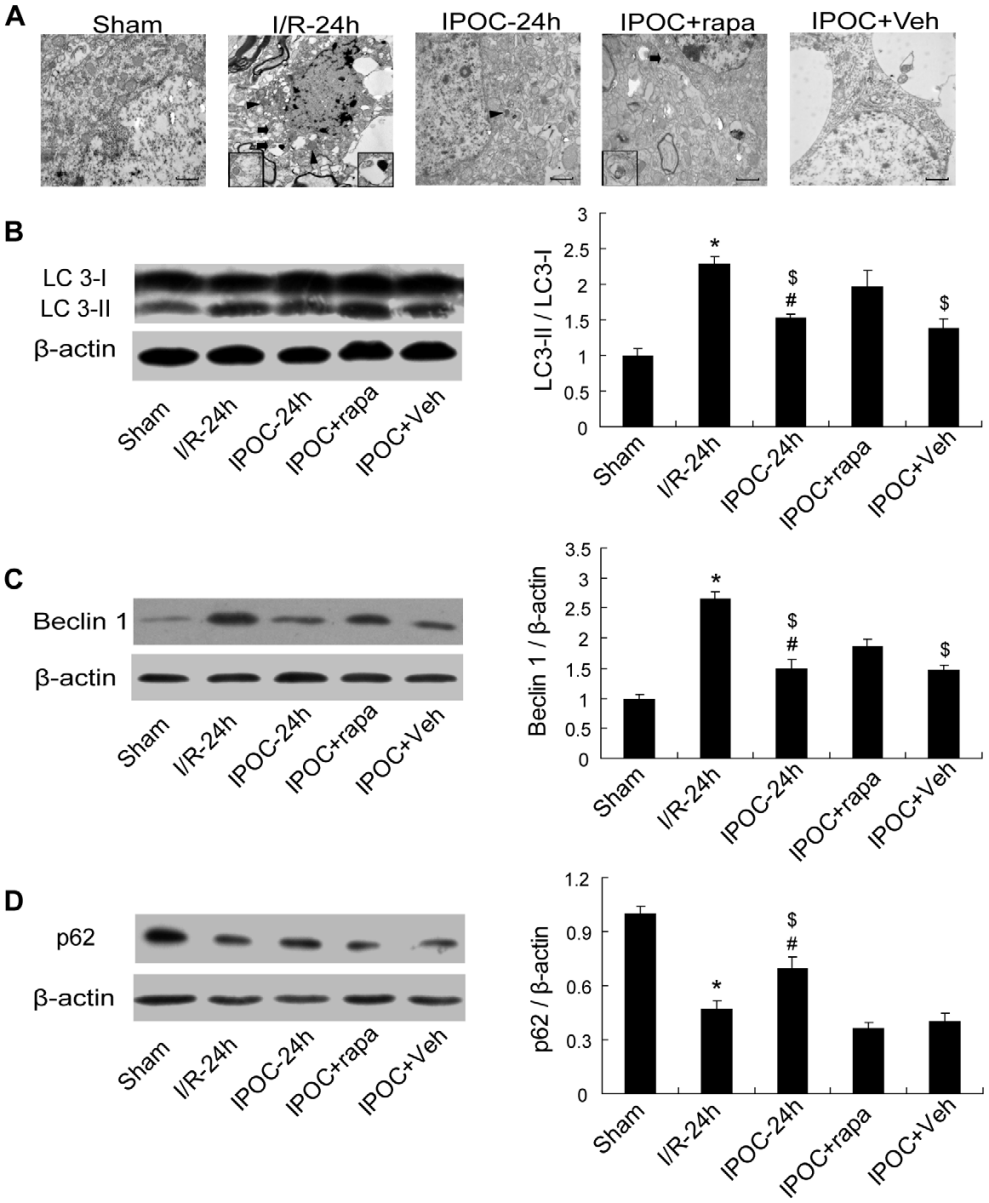
Rapamycin partially attenuated the autophagic inhibition effects induced by IPOC. (A) The formation of autophagosomes in cerebral cortex in the Sham, I/R-24 h, IPOC-24 h, IPOC+rapa and IPOC+Veh groups at 24 h after ischemia. Rats were perfused with 4% PFA and 1% glutaradehyde and processed for electron microscopic examination. The photomicrographs showed represent samples taken from 3 rats in each group. Arrows indicate autophagosomes. Arrowheads indicate lysosomes. Insets in the pictures were enlarged autophagosomes taken from the areas indicated by arrows. Scale bars: 1 µm. (B–D) Western blot analysis of the expressions of LC3, Beclin 1 and p62 in the penumbra in the Sham, I/R-24 h, IPOC-24 h, IPOC+rapa and IPOC+Veh groups at 24 h after ischemia. Rapamycin upregulated the expressions of LC3/Beclin 1 and downregulated p62 when compared with that in IPOC group. Levels of β-actin protein were used as the loading control. n = 5 for each group. *p<0.05 vs. the Sham group; ^#^ *p*<0.05 vs. I/R-24 h group; ^$^ *p*<0.05 vs. IPOC+rapa group.

Twenty-four hours after ischemia, morphological changes of neurons were observed by transmission electron microscopy (TEM) in the ischemic penumbra of cerebral cortex (Figure 4A). In the Sham group, the cortical neurons appeared normal, with relatively healthy-looking nuclei, mitochondria, endoplasmic reticulum and a few double membrane-bound compartments. After ischemia, the cortical neurons in I/R-24 h group were vacuolated with disrupted cell structure and shrunken nuclei. The mitochondria were visibly swollen with vacuolated and disorganized cristae. The endoplasmic reticulum were dilated and fragmented, with some ribosomes detached from the rough ones. Many damaged neurons displayed apoptotic or necrotic morphological features: condensation of chromatin, shrinkage of cells, and rupture of cell membranes. Meanwhile, increased autophagosomes and autolysosomes with cytoplasmic material and enhanced electron density were observed in damaged neurons as well, implying the involvement of autophagosomal/lysosomal component in ischemia-induced cell death. In the IPOC+rapa group, increased autophagosomes were also found. Some neurons were apparently injured in morphology with disrupted cell structure, but the formation of cytosolic vacuoles decreased when compared with the I/R-24 h group. Comparatively, the IPOC-24 h or IPOC+Veh group revealed moderately swollen mitochondria and dilated endoplasmic reticulum, with fewer autophagosomes and autolysosomes, and less severe cell damage than that in I/R-24 h or IPOC+rapa group.

The electron-microscopic morphology prompted us to further evaluate the association between autophagy inhibition and postconditioning treatment following ischemia. To confirm rapamycin-induced autophagy, the expressions of LC3, Beclin 1 and p62 were examined in Sham, I/R, IPOC, IPOC+rapa and IPOC+Veh groups at 24 h postischemia. In rats treated with rapamycin plus postconditioning, the ratio of LC3-II/LC3-I and the expression of Beclin 1 increased significantly as compared with that in the IPOC group or IPOC+Veh group, but still lower than the levels in I/R-24 h group (Figure 4B–C; *p<0.05 vs. the Sham group; ^#^ *p*<0.05 vs. I/R-24 h group; ^$^ *p*<0.05 vs. IPOC+rapa group). The expression of p62 displayed a downregulation after rapamycin administration with IPOC (Figure 4D; *p<0.05 vs. the Sham group; ^#^ *p*<0.05 vs. I/R-24 h group; ^$^ *p*<0.05 vs. IPOC+rapa group), implicating that IPOC-induced inhibition of autophagy may be partially attenuated by rapamycin.

**Figure 5:**
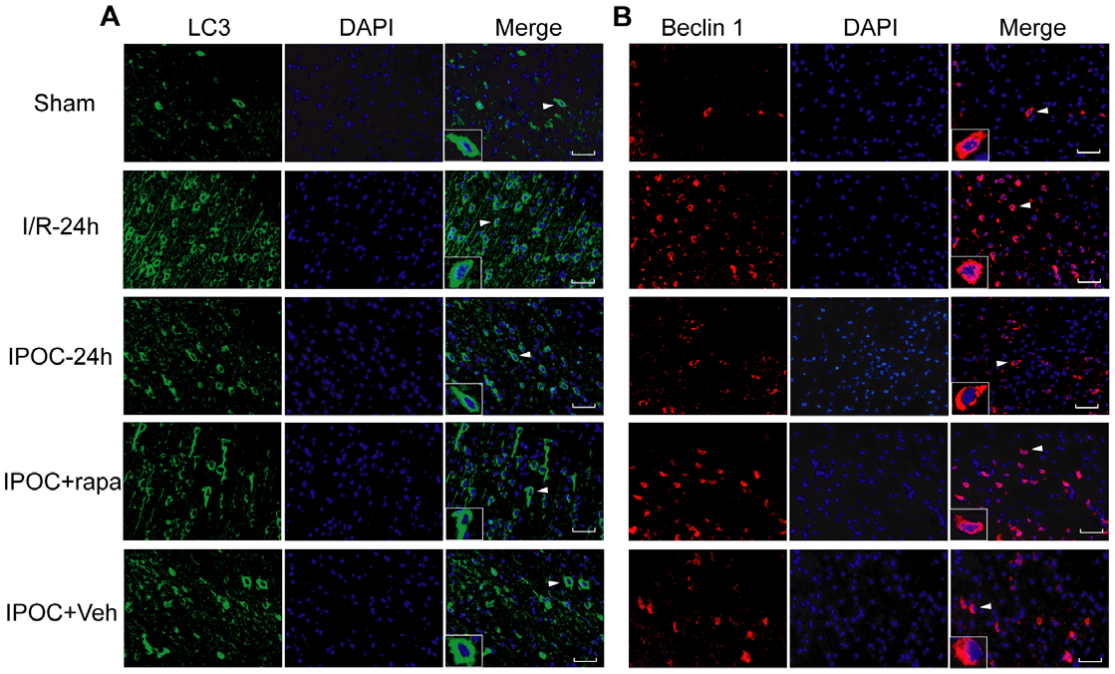
Cellular localization of LC3 and Beclin 1 after postconditioning. (A) Cellular localization of LC3 after postconditioning. (B) Cellular localization of Beclin 1 after postconditioning. Rats were fixed with 4% PFA, dehydrated in alcohol, and the brain sections of the ipsilateral hemisphere were double-labeled with the anti-LC3 antibody (green) and DAPI (blue) or anti-Beclin 1 antibody (red) and DAPI (blue). The LC3 immunoreactivity was weaker in IPOC-24 h and IPOC+Veh groups than that in I/R-24 h group. When rapamycin was administrated before postconditioning, the LC3 immunoreactivity was relatively enhanced, but weaker than that in I/R-24 h group. Similarly, rapamycin plus postconditioning treatment increased Beclin 1 immunoreactivity, but not as strong as that in I/R-24 group. n = 3 in each group. Brain sections were viewed under a fluorescence microscopy. Insets in the pictures were amplified immunofluorecence staining taken from the areas indicated by arrowheads. Scale bar: 400 µm.

To confirm the above observations, the immunofluorescence of LC3 and Beclin1 in the cerebral cortex of ipsilateral hemisphere was examined. In double staining, LC3 and DAPI showed a granular, homogeneous pattern almost exclusively in the region of the perikaryon (Figure 5A). In the Sham group, LC3 immunoreactivity was low. Twenty-four hours after ischemia, the immunoreactivity increased robustly in I/R-24 h group in the surrounding area of the ischemic core, however, it declined greatly at the corresponding regions after postconditioning treatment. Whereas in the IPOC+rapa group, the number of cells displaying increased LC3 immunostaining was more than that in IPOC-24 h and IPOC+Veh groups, but fewer than that in I/R-24 group. Similarly, Beclin 1 displayed granular staining in the perikaryal region of cells and a marked induction of immunoreactivity in I/R-24 h group, but not in IPOC-24 h and IPOC+Veh group, whose immunoreactivity was relatively weak (Figure 5B). When rapamycin was administered before postconditioning, the immunostaining of Beclin 1 was stronger than that in IPOC-24 h and IPOC+Veh groups.

Taken together, the results from the western blot analysis and the morphological changes presented in TEM and immunofluorescence studies suggest that inhibition of autophagic pathway contributes to the neuroprotection of IPOC.

### 3-MA mimics the neuroprotection induced by IPOC

**Figure 6:**
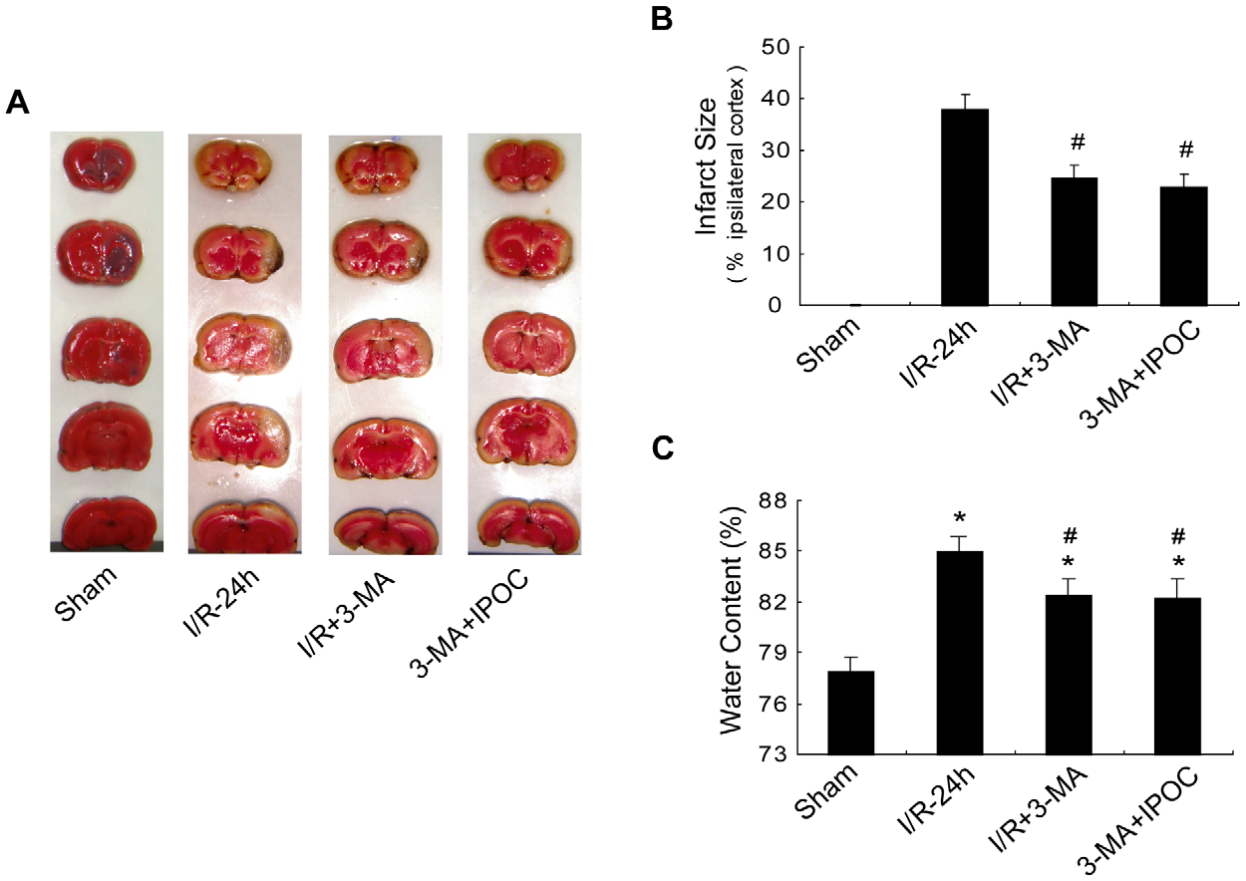
Treatment with 3-MA alone or combined with IPOC reduced brain infarction and water content after ischemia. Rats were treated with an i.c.v. injection of 600 nmol 3-MA or combined with IPOC at the onset of reperfusion, then followed by 24 h reperfusion. (A) Representative TTC staining from rat brains in the Sham, I/R-24 h, I/R+3-MA and 3-MA+IPOC groups. (B) Quantification of infarct size from each group at 24 h after ischemia. (C) Quantification of water content from each group at 24 h after ischemia. 3-MA alone or combined with IPOC reduced infarct size and mitigated brain edema induced by cerebral ischemia. n = 8 in each group. *p<0.05 vs. the Sham group; ^#^ *p*<0.05 vs. I/R-24 h group.

**Figure 7:**
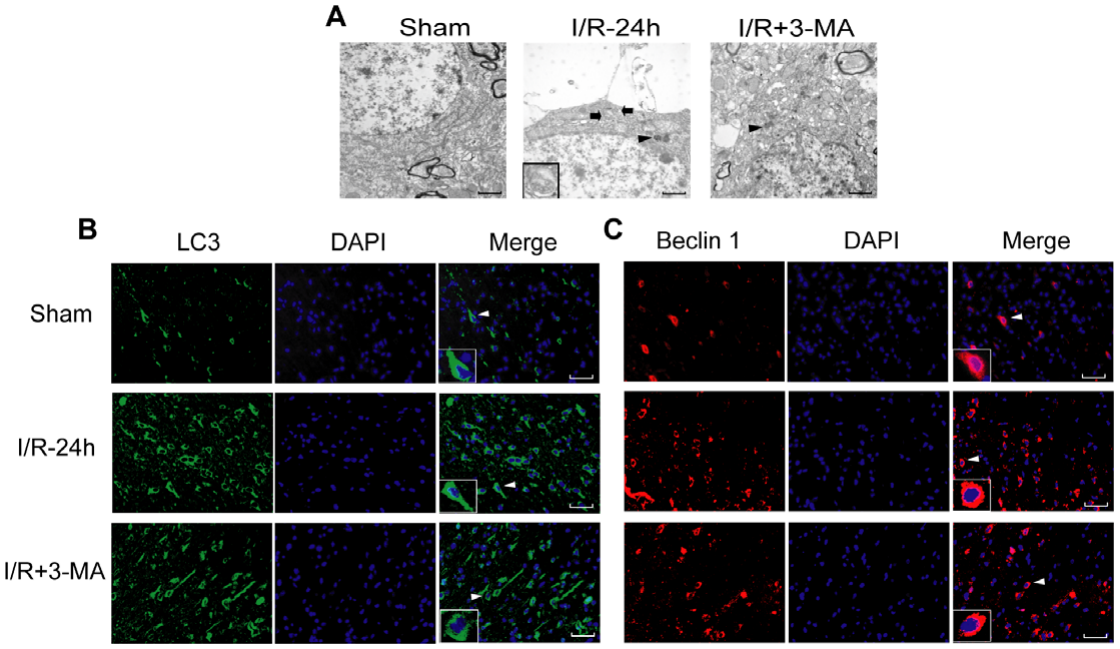
3-MA inhibited the formation of autophagosomes and reduced the immunoreactivity of LC3 and Beclin 1. (A) Ultrastructural morphology of cerebral cortex in the Sham, I/R-24 h and I/R+3-MA groups at 24 h after ischemia under transmission electron microscope. Rats were perfused with 4% PFA and 1% glutaradehyde and processed for electron microscopic examination. The photomicrographs showed represent samples taken from 3 rats in each group. Arrows indicate autophagosomes. Arrowheads indicate lysosomes. Insets in the pictures were enlarged autophagosomes taken from the areas indicated by arrows. Scale bars: 1 µm. (B–C) The immunofluorescence of LC3 and Beclin1 in the cerebral cortex of ipsilateral hemisphere. The coronal sections were double-labeled with the anti-LC3 antibody (green) and DAPI (blue) or anti-Beclin 1 antibody (red) and DAPI (blue). Both the immunoreactivity of LC3 and Beclin 1 elevated robustly in I/R-24 h group, however, it declined greatly at the corresponding regions after 3-MA treatment. n = 3 in each group. Brain sections were viewed under a fluorescence microscopy. Insets in the pictures were amplified immunofluorecence staining taken from the areas indicated by arrowheads. Scale bar: 400 µm.

**Figure 8:**
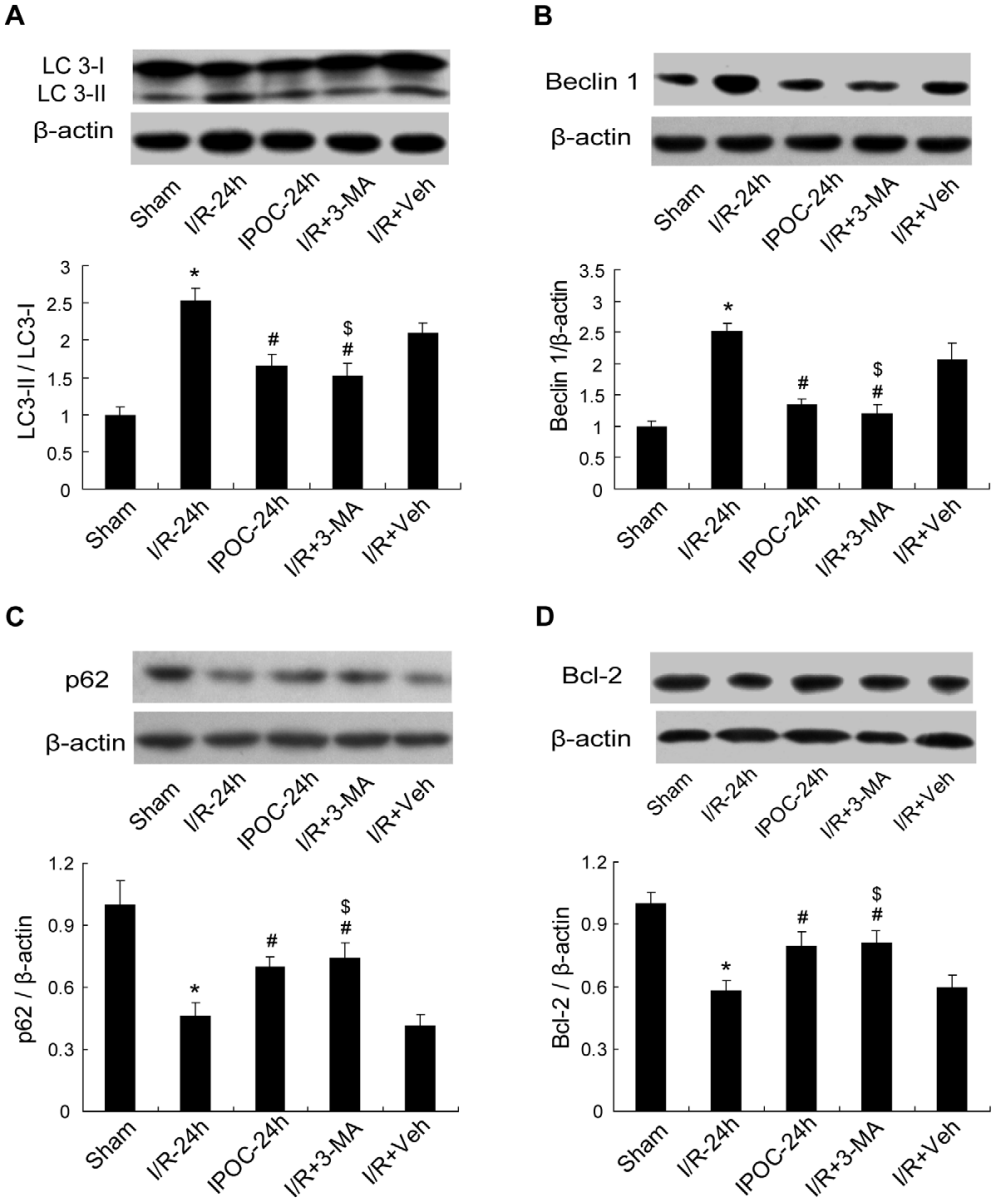
The changes of expressions of LC3, Beclin 1, p62 and Bcl-2 after 3-MA treatment. (A–C) The changes of LC3, Beclin 1 and p62 after the treatment of 3-MA. 3-MA downregulated the ratio of LC3-II/LC3-I and the expression of Beclin 1, and upregulated the expression of p62 as compared with that in I/R-24 h or I/R+Veh group. (D) The expression of Bcl-2 after the treatment of 3-MA. The expression of Bcl-2 decreased at 24 h postischemia, however, IPOC or treatment with 3-MA partially resulted in the recovery of Bcl-2. Levels of β-actin protein were used as the loading control. n = 5 for each group. *p<0.05 vs. the Sham group; ^#^ *p*<0.05 vs. I/R-24 h group; ^$^ *p*<0.05 vs. I/R+Veh group.

If autophagy inhibition is invoved in the neuroprotection of IPOC, then whether autophagy inhibitor 3-MA could mimic some neuroprotective effects induced by IPOC still remains unknown. As previously discovered, i.c.v. of 150 to 600 nmol 3-MA has protective effects against focal cerebral ischemia [20], [24], then a dose of 600 nmol 3-MA was used for the current study. 3-MA administered before reperfusion significantly reduced infarct size and abolished the ischemia-induced increase in brain water content. In addition, the infarct size in 3-MA+IPOC group was smaller than that in 3-MA alone group, however, there was no significant difference between the two groups. Simultaneously, the results of water content measurement showed no significant difference between the two groups as well. (Figure 6A–C; *p<0.05 vs. the Sham group; ^#^ *p*<0.05 vs. I/R-24 h group). The morphological changes under TEM showed that after 3-MA treatment, though swollen mitochondria, dilated endoplasmic reticulum and cytoplasmic vacuoles still existed in some neurons of the ischemic cortex, autophagosomes were hardly observed and the cell damage seemed much less than that in I/R-24 h group (Figure 7A). The immunofluorescence test indicated that the immunoreactivity of both LC3 and Beclin 1 declined strongly in the I/R+3-MA group in the penumbra as compared with that in the I/R-24 h group (Figure 7B–C). Moreover, after 3-MA treatment, the ratio of LC3-II/LC3-I and the protein level of Beclin 1 decreased while p62 increased (Figure 8A–C; *p<0.05 vs. the Sham group; ^#^ *p*<0.05 vs. I/R-24 h group; ^$^ *p*<0.05 vs. I/R+Veh group). The results implicated that the autophagy inhibitor 3-MA, when administrated at the onset of reperfusion, could ameliorate the ischemic injuries and inhibit autophagic induction, and that 3-MA may mimic some protective effects of IPOC to elicit neuroprotection against focal cerebral ischemia. In addition, the expression of Bcl-2 in the penumbra was detected. The immunoblotting result showed that the protein level of Bcl-2 was lower than that in the sham-operated rats at 24 h postischemia (Figure 8D; *p<0.05 vs. the Sham group). However, IPOC or treatment with 3-MA before reperfusion could partially result in the recovery of Bcl-2 (Figure 8D; ^#^ *p*<0.05 vs. I/R-24 h group; ^$^ *p*<0.05 vs. I/R+Veh group), indicating that upregulation of Bcl-2 is involved in the neuroprotective effects elicited by IPOC and 3-MA in this focal cerebral ischemic model.

## Discussion

In the present study, we established a focal cerebral ischemic model with permanent MCA occlusion plus transient CCA occlusion and found that autophagy was activated in the penumbra at various time intervals following ischemia. IPOC, performed at the onset of reperfusion, significantly decreased infarct size, mitigated brain edema, and simultaneously, attenuated the induction of autophagy at 24 h after ischemia. Whereas all the neuroprotective effects induced by IPOC were partially reversed by the autophagy inducer, rapamycin. Conversely, 3-MA inhibited autophagy induction, upregulated the expression of anti-apoptotic protein Bcl-2, and attenuated the ischemic insults to an extent comparable to IPOC. While 3-MA combined with IPOC didn't reinforce the neuroprotective effect of each other. These results suggest that the inhibition of autophagic pathway partially underlies the mechanism of IPOC-induced tolerance to cerebral ischemia.

Over the past several decades, failed clinical trials of exogenously administered drugs as potential neuroprotectants have enhanced the ongoing search to identify endogenously modulated mechanisms that might be harnessed as neuroprotectants in stroke treatment [26], [27]. In this attempt, ischemic preconditioning (IPC) and IPOC represent two potent strategies in modulating ischemic damage. IPC is the phenomenon whereby a brief non-injurious episode of ischemic stress renders the organ resistant to a subsequent lethal ischemic insult [26]. Although extensive research has demonstrated that IPC reduces cerebral ischemic damage, it is clinically feasible only when the occurrence of stroke is predictable. Postconditioning, by definition, performed after ischemia and just at the onset of reperfusion, has translational relevance to reperfusion and thrombolitic therapy in acute cerebral ischemia. Some earlier studies reported that autophagy activation is associated with the mechanism of IPC and inhibition of autophagy abrogates the protective effects of IPC [25], [28]. Whether autophagy is involved in the tolerance induced by IPOC to cerebral ischemia still remains unknown. This formalized hypothesis makes it more likely that our research will lead to meaningful results.

At present, we tested our hypothesis in a focal cerebral ischemic postconditioning model with permanent MCA occlusion plus transient CCA occlusion. For the bilateral CCAs release allows partial reperfusion, which models clinical cases in which recanalization is incomplete [2]. Previous studies have demonstrated autophagy activation in the intraluminal thread model of middle cerebral artery occlusion (MCAO) in rats and mouse [19]–[21]. Consistently, we observed autophagy stimulation in this CCA occlusion plus permanent MCA occlusion model with the upregulation of LC3/Beclin 1 and downregulation of p62, further confirming autophagy activation in focal cerebral ischemia. However, after postconditioning, the induction of LC3 and Beclin 1 was inhibited and the reduction of p62 was reversed at 24 h postischemia, implying that IPOC may be correlated with autophagy inhibition and that IPOC can protect neuronal survival through inhibiting autophagic pathway. In recent years, some studies have suggested that autophagy is involved in the regulation of neuronal death following cerebral ischemia and that postischemic treatment of neonatal cerebral ischemia should target autophagy directly [20], [22], [29], [30]. For the first time, we found that the neuroprotective effects of IPOC may be associated with autophagy inhibition as well. To further evaluate the contribution of the autophagic mechanism to IPOC-induced neuroprotection and to assess whether the neuroprotective effect could be abolished by pharmacological interactions, rapamycin was applied before postconditioning. Rapamycin, a macrolide antibiotic, is often used as autophagy inducer for its inhibition of the mammalian target of rapamycin (mTOR), which negatively controls autophagy [31]. The data indicated that rapamycin alone was not neuroprotective, but attenuated the neuroprotective effects of IPOC. As rapamycin plus postconditioning treatment increased infarct size, aggregated brain edema, upregulated the formation of autophagosomes and enhanced the autophagic activity when compared with IPOC-only treatment, suggesting that rapamycin attenuates the neuroprotection of IPOC and inhibition of autophagy is involved in the mechanism of IPOC. In the past, some studies found that rapamycin alone was not neuroprotective, but abolished the neuroprotective effects of FK506 when coadministered in vivo [32], [33]. Labrande et al. further reported rapamycin was unable to protect neuronal cells from oxygen-glucose deprivation but cancelled the neuroprotective effect of tacrolimus in vitro [34]. Our findings are consistent with previous studies of myocardial ischemia, in which autophagic cardiomyocytes decreased significantly after postconditioning but rapamycin completely blocked the infarct size reduction of IPOC or isoflurane-elicited pharmacological postconditioning [35]–[38]. Furthermore, we explored if 3-MA could mimic the protective effects of IPOC. 3-MA, a widely used inhibitor of autophagy, suppresses autophagy by inhibiting Class III phosphatidylinositol 3-kinase (PI3K), whose activity is required for the membrane dynamics involved in autophagic vesicle trafficking [39]. In this study, 3-MA administrated before reperfusion ameliorated ischemic injuries, downregulated the autophagic activity and increased the protein level of Bcl-2, thus exerted neuroprotective effects compatible with IPOC. In addition, 3-MA combined with IPOC significantly reduced ischemic injuries as well, but didn't reinforce the neuroprotective effect of each other, implicating inhibiting autophagic pathway at least partially underlies the mechanism of IPOC-induced tolerance to cerebral ischemia. The neuroprotective effects of 3-MA were supported by the findings that 3-MA elicited a major protective effect against focal cerebral ischemia when applied immediately after ischemia or even 3 h after reperfusion in adult and neonatal rats [20], [29]. More recently, Cui et al. further proved that inhibiting autophagy activation and maturation can reduce I/R injury and improve cell survival in vivo and in vitro [40]. In this study, we suggest that the neuroprotective effects elicited by IPOC are partially attenuated by rapamycin, whereas 3-MA can mimic the neuroprotective effects of IPOC.

However, several studies have reached the opposite conclusion that autophagy activation is associated with protective effects conferred by IPC and that autophagy inhibition may aggregate ischemic insults [21], [23], [25]. Sheng et al. [25] have demonstrated that autophagy activation was involved in the neuroprotective mechanism of IPC and that rapamycin pretreatment produced beneficial preconditioning-like effects, while pretreatment with 3-MA suppressed the neuroprotective effects of IPC. Causes for the inconsistency are unclear. The different models of ischemia, the durations of the ischemic insults and the different ages of animals are offered as possible explanations. It is reported that autophagy is more pronounced in adult mice than in neonatal mice subjected to hypoxia-ischemia injury and autophagic cell death may be more significant in mature animals [41]. Furthermore, although IPC and IPOC are both beneficial to neuronal survival, IPOC is conducted after reperfusion thus alters events after ischemia but IPC is administered prior to ischemia. The possible mechanisms and the conducted timing are different between the two maneuvers [27]. In addition, the timing of rapamycin and 3-MA administration might be crucial to the discrepancies. In the process of IPC, 3-MA applied at the beginning of ischemia aggregated ischemic injuries and rapamycin pretreatment produced a potent preconditioning-like effect [25]. Therefore, when rapamycin is administered after ischemia, the level of autophagy will be further raised, hypothetically resulting in excessive autophagy induction. Conversely, 3-MA applied after ischemia or even 3 h after reperfusion could reduce infarct size and attenuate the ischemic injuries, which was supported by the present sturdy as well as the findings from other laboratories [20], [29]. Paradoxically, it is also reported that 3-MA administered before ischemia provided time-dependent protection against hippocampal neuronal death and even reduced astrocyte damage after ischemia in vivo and in vitro [24], [42]. Therefore, the timing of administration is just one determinant of protective effects. In line with this hypothesis is the finding that rapamycin pretreatment blocked the cardioprotection mediated by IPC in myocardial ischemia [43]. That is to say, the association of autophagy with the neuroprotective effects of IPC and IPOC is highly contextual. It is still not clear which level of autophagy decides between cell survival and death in cerebral ischemia. In the present study, we found that inhibition of autophagic pathway contributed to IPOC-elicited neuroprotection against focal cerebral ischemia and that autophagy inhibition after ischemia may represent a potential neuroprotective mechanism. Further work is needed to clarify autophagy-mediated signaling pathway through which IPOC induces neuroprotection.

In addition, we observed that 3-MA and IPOC treatment both downregulated the expressions of LC3 and Beclin 1, upregulated p62, and reversed the decline of Bcl-2, suggesting a cross-talk between autophagy and apoptosis in postconditioning. Bcl-2 is one of the most abundant anti-apoptotic proteins in Bcl-2 family members. In the past several years, the biological effects of Bcl-2 have been largely attributed to its effects on the apoptotic pathway. Previous studies have indicated that IPOC could upregulated Bcl-2 expression, reduced cytochrome c release to the cytosol, and caspase-3 activity thus inhibited apoptosis after cerebral ischemia [2], [7]. Recently, it is reported that Bcl-2 anti-apoptotic family members also target the autophagic pathway [15]. Bcl-2 could target Beclin 1-dependent autophagic pathway via its inhibitory interaction with the autophagic marker Beclin 1. Beclin 1 possesses a BH3-only domain that dictates its physical and functional interaction with the BH3 binding groove of multi-domain proteins of the Bcl-2 family. Therefore, Bcl-2 can constitutively interact with Beclin1 and function as a brake on levels of autophagy and autophagic cell death. Our findings suggest there is a crosstalk between autophagy and apoptosis in Bcl-2 levels in the process of IPOC, however, the cause-effect relationship between Bcl-2 and autophagy during IPOC needs to be further studied.

In summary, the current study demonstrates that the neuroprotection induced by IPOC against ischemic injury is mediated, at least partly, by inhibiting of autophagic pathway. The autophagic inhibitor 3-MA can be used to reduce damage of fatal cerebral ischemia, which may provide novel strategies for clinical treatment of ischemic stroke.

## Materials and Methods

Male Sprague-Dawley rats weighing 300–400 g were housed according to the Guide for the Care and Use of Laboratory Animals. All procedures were approved by the Ethics Committee for the Use of Experimental Animals at Nanjing Medical University (permit number: NYLL2009-0005). The rats were housed in individual cages under controlled conditions, with a 12-hour light/dark cycle, a temperature of 24±2°C and free access to food and water. All surgery was performed under anesthesia and all efforts were made to minimize the suffering of the animals.

### Experimental design

**Figure 9:**
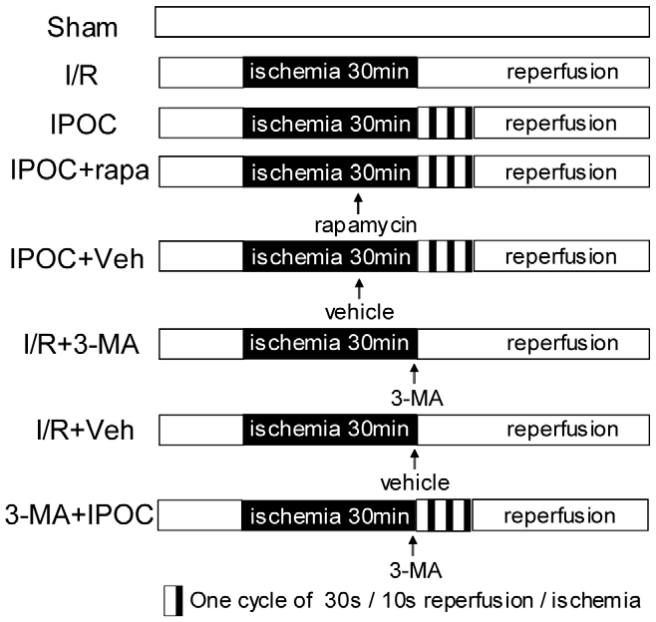
Experimental protocol used to evaluate the role of autophagy in IPOC- induced neuroprotection. Sham: sham-operated rats; I/R: rats subjected to 30 min of bilateral CCAs occlusion plus permanent distal MCA occlusion followed by reperfusion; IPOC: rats subjected to ischemia plus postconditioning of 3 cycles of 30 sec reperfusion and 10 sec occlusion; IPOC+rapa: rats treated with an i.c.v. injection of 35 pmol rapamycin 15 min before postconditioning; IPOC+Veh: rats received the same volume of vehicle 15 min before postconditioning; I/R+3-MA: rats subjected to ischemia were treated with 600 nmol 3-MA at the onset of reperfusion; I/R+Veh: rats treated with the same volume of vehicle at the onset of reperfusion; 3-MA+IPOC: rats subjected to ischemia were treated with 600 nmol 3-MA plus postconditioning at the onset of reperfusion. IPOC = ischemic postconditioning; rapa = rapamycin; 3-MA = 3-methyl-adenine; Veh = vehicle.

All animals were randomly assigned to one of eight groups, as shown in Figure 9:

1. Sham group: animals were undergone the surgical procedures but without ischemia;
2. I/R group: rats were subjected to 30 min of ischemia followed by 1, 6, 12, 24, and 48 h of reperfusion;
3. IPOC group: rats were subjected to ischemia plus postconditioning, and then followed by 6 h and 24 h of reperfusion;
4. IPOC+rapa group: rats were treated with an i.c.v. injection of 35 pmol rapamycin (Sigma, R0395) 15 min before IPOC as described previously [44], then followed by 24 h of reperfusion.
5. IPOC+Veh group: rats received an i.c.v. injection of the same volume of vehicle 15 min before postconditioning and followed by 24 h of reperfusion;
6. I/R+3-MA group: rats subjected to ischemia were treated with an i.c.v. injection of 600 nmol 3-MA (Sigma, M9281) at the onset of reperfusion, then followed by 24 h of reperfusion.
7. I/R+Veh group: rats subjected to ischemia were treated with an i.c.v. injection of same volume of vehicle at the onset of reperfusion and followed by 24 h of reperfusion.
8. 3-MA+IPOC group: rats subjected to ischemia were treated with an i.c.v. injection of 600 nmol 3-MA at the onset of reperfusion, then mamuplated with postcontioning, and then followed by 24 h of reperfusion.

### Focal cerebral ischemia

Rats were anesthetized with 10% chloral hydrate (350 mg/kg, i.p.) and the core body temperature was kept at 37°C during the whole experiment. Focal cerebral ischemia was conducted as described previously [2]. In brief, the bilateral CCAs were separated and the distal MCA was exposed. The bilateral CCAs were occluded first and then the distal MCA was cauterized permanently above the rhinal fissure 2 min later. After 30 minutes of CCAs occlusion plus MCA cauterization, the CCAs were released to allow partial reperfusion through collateral blood flow. For postconditioning, the CCAs were released for 30 sec followed by 10 sec reocclusion, repeated for three cycles, and then allowed reperfusion.

For the i.c.v. injection, some anesthetized rats were placed in a stereotaxic apparatus and a burr hole was drilled into the bone 1.4 mm lateral and 0.8 mm posterior to bregma over the left hemisphere. Using a microinjector, rapamycin, 3-MA or vehicle was infused into the ventricular space ipsilateral to ischemia. Rapamycin was first dissolved in ethanol and then diluted in normal saline solution (the final ethanol concentration <2%). 3-MA was dissolved in normal saline by heating solution to 60–70°C immediately before treatment. The dosages of rapamycin and 3-MA were selected according to previous studies [20], [24], [25].

### The measurement of infarct size measurement and brain edema

Twenty-four hours after ischemia, rats (n = 8 for each group) were anesthetized and decapitated. Their brains were rapidly removed and sliced into five 3-mm-thick coronal sections and immersed in 2% 2,3,5-triphenyltetrazolium chloride (TTC) for 30 min at 37°C, followed by overnight immersion in 4% paraformaldehyde (PFA). The percentage of infarct cortex was normalized to the contralateral non-ischemic cortex and expressed according to the formula: [contralateral cortex−(ipsilateral cortex−infarct cortex)]/contralateral cortex×100%. For edema measurement, the wet-dry weight method with slight modification was employed. The total TTC stained brains were weighed for wet weight, baked at 90±2°C for 1 week, and weighed again for dry weight. Water content in the brain tissues were calculated as: (wet weight-dry weight)/wet weight×100%.

### Western blotting assay

Rats were decapitated and the brains were harvested to prepare ipsilateral penumbra tissue samples (n = 5 for each time point in each group). The ischemic penumbra was determined according to the methods described previously [2]. Total proteins were extracted and the concentration was determined by the Bradford method. An equal amount of protein (30–50 µg) was loaded in each lane and subjected to 10–15% sodium dodecyl sulfate-polyacrylamide gel electrophoresis (SDS-PAGE). Separated proteins were then transferred to nitrocellulose membranes. Afterwards, the membranes were blocked with 5% skimmed milk in Tris-buffered saline-Tween 20 (TBST) for 2 h and incubated with the primary antibodies against LC3 (1∶1000, Sigma, L7543), Beclin 1 (1∶1000, Cell Signaling Technology, #3738), p62 (1∶1000, Enzo Life Science, BML-PW9860), Bcl-2 (1∶1000, Cell Signaling Technology, #2876) and β-actin (1∶500, Santa Cruz Biotechnology, sc-130301) overnight at 4°C. After rinsing with TBST, the membranes were incubated with horseradish peroxidase (HRP)-conjugated secondary antibody (1∶10000, Zhongshan goldenbridge, ZB-2301) for 2 h at room temperature and developed with an enhanced chemiluminescence system (ECL kit, Thermo). The films were scanned and the optical density was quantified using Biosens Gel Imaging System (Biosens, SC810), and then the optical density ratio was normalized to sham.

### Transmission electron microscopic examination

Twenty-four hours after ischemia, rats were deeply anesthetized and transcardially perfused with 0.1 M phosphate buffered (PBS, pH 7.4), followed by 4% PFA and 1% glutaraldehyde (n = 3 for in each group). The parietal lobe cortex in the ischemic penumbra selected for analysis was cut into small sections and kept overnight in the same fixative. The selections were then immersed in 1% osmium tetroxide for 2 h, dehydrated in graded ethanol and then embedded in epoxy resin. Afterwards, the selections were cut into ultrathin sections (60–70 nm) with an ultramicrotome and post-stained with uranyl acetate and lead citrate, and subsequently examined under a transmission electron microscope (JEOL, JEM-1010).

### Immunofluorescence

Twenty-four hours after ischemia, rats were deeply anesthetized and transcardially perfused with PBS, followed by 4% PFA dissolved in PBS (n = 3 for each group). Brains were removed and postfixed in 4% PFA overnight. After dehydrated in alcohol, the brains were embedded in paraffin and cut into 4–5 µm sections. Sections were deparaffinized, hydrated in distilled water, treated with 3% H_2_O_2_ for 10 min to remove residual peroxidase activity, and rinsed again with PBS. Afterwards, sections were permeabilized with 0.1% TritonX-100 for 10 min, blocked with 10% normal goat serum for 2 h at room temperature and incubated in the primary antibodies against LC3 (1∶400, Sigma, L7543) or Beclin 1 (1∶500, Abcam, ab62472) at 4°C for 24 h. The sections were rinsed with PBS and sequentially incubated respectively with FITC conjugated anti-rabbit IgG (1∶200, Zhongshan goldenbridge, ZF-0311) or TRITC conjugated anti-rabbit IgG (1∶200, Zhongshan goldenbridge, ZF-0316) in a humidified container for 1 h at 37°C. Then the sections were further incubated with 0.5 µg/ml 4,6-diamidino-2-phenylindole (DAPI) for 10 min. After that, the sections were washed with PBS and sealed with a coverslip. The slides were analyzed with a fluorescence microscopy (Olympus, BX51).

### Statistics analysis

The statistical analyses were performed with SPSS 16.0 and the values were carried out by one-way analysis of variance (ANOVA). The intergroup comparisons (post-hoc analysis) among the data with equal variances were carried out with the least significant difference (LSD) method, whereas Dunnett T3 method was used for the data with unequal variances. All results were expressed as mean±SEM and were examined for the homogeneity of variance. Statistical significance was defined as *p*<0.05.

## Supporting Information

Figure S1 **TC staining and brain edema measurement from rat brains in the Sham, I/R-24 h and I/R+rapa groups.** Rats were treated with an i.c.v. injection of 35 pmol rapamycin at the onset of reperfusion, and then followed by 24 h reperfusion. (A) Representative infarcts stained with TTC in the Sham, I/R-24 h and I/R+rapa groups 24 h after stroke. (B) Quantification of infarct size from each group at 24 h after ischemia. (C) Quantification of water content from each group at 24 h after ischemia. The results of TTC and water edema measurement showed there was no significant difference between the ischemia-only and rapamycin-treated rats at 24 h after ischemia. n = 8 for each group. *p<0.05 vs. the Sham group.
(TIF)

## References

[1] Donnan GA, Fisher M, Macleod M, Davis SM (2008) Stroke. Lancet 371: 1612–1623. 18468545 10.1016/S0140-6736(08)60694-7

[2] Zhao H, Sapolsky RM, Steinberg GK (2006) Interrupting reperfusion as a stroke therapy: ischemic postconditioning reduces infarct size after focal ischemia in rats. J Cereb Blood Flow Metab 26: 1114–1121. 16736038 10.1038/sj.jcbfm.9600348

[3] Wang JK, Yu LN, Zhang FJ, Yang MJ, Yu J, et al (2010) Postconditioning with sevoflurane protects against focal cerebral ischemia and reperfusion injury via PI3K/Akt pathway. Brain Res 1357: 142–151. 20705063 10.1016/j.brainres.2010.08.009

[4] Danielisová V, Gottlieb M, Némethová M, Kravčuková P, Domoráková I, et al (2009) Bradykinin postconditioning protects pyramidal CA1 neurons against delayed neuronal death in rat hippocampus. Cell Mol Neurobiol 29: 871–878. 19259804 10.1007/s10571-009-9369-3 PMC11505757

[5] Leconte C, Tixier E, Freret T, Toutain J, Saulnier R, et al (2009) Delayed hypoxic postconditioning protects against cerebral ischemia in the mouse. Stroke 40: 3349–3355. 19628803 10.1161/STROKEAHA.109.557314

[6] Gao XW, Zhang HF, Takahashi T, Hsieh J, Liao J, et al (2008) The Akt signaling pathway contributes to postconditioning's protection against stroke; the protection is associated with the MAPK and PKC pathways. J Neurochem 105: 943–955. 18182053 10.1111/j.1471-4159.2008.05218.x PMC2746404

[7] Xing BZ, Chen H, Zhang M, Zhao DM, Jiang R, et al (2008) Ischemic postconditioning inhibits apoptosis after focal cerebral ischemia/reperfusion injury in the rat. Stroke 39: 2362–2369. 18583563 10.1161/STROKEAHA.107.507939

[8] Staat P, Rioufol G, Piot C, Cottin Y, Cung TT, et al (2005) Postconditioning the human heart. Circulation 112: 2143–2148. 16186417 10.1161/CIRCULATIONAHA.105.558122

[9] Ivanes F, Rioufol G, Piot C, Ovize M (2011) Postconditioning in acute myocardial infarction patients. Antioxid Redox Signal 14: 811–820. 20578963 10.1089/ars.2010.3354

[10] Mizushima N (2007) Autophagy: process and function. Genes Dev 21: 2861–2873. 18006683 10.1101/gad.1599207

[11] Yorimitsu T, Nair U, Yang ZF, Klionsky DJ (2006) Endoplasmic Reticulum Stress Triggers Autophagy. J Biol Chem 281: 30299–30304. 16901900 10.1074/jbc.M607007200 PMC1828866

[12] Sarkar S, Perlstein EO, Imarisio S, Pineau S, Cordenier A, et al (2007) Small molecules enhance autophagy and reduce toxicity in Huntington's disease models. Nat Chem Biol 3: 331–338. 17486044 10.1038/nchembio883 PMC2635561

[13] Kabeya Y, Mizushima N, Ueno T, Yamamoto A, Kirisako T, et al (2000) LC3, a mammalian homologue of yeast Apg8p, is localized in autophagosome membranes after processing. EMBO J 19: 5720–5728. 11060023 10.1093/emboj/19.21.5720 PMC305793

[14] Liang XH, Jackson S, Seaman M, Brown K, Kempkes B, et al (1999) Induction of autophagy and inhibition of tumorigenesis by beclin 1. Nature 402: 672–676. 10604474 10.1038/45257

[15] Pattingre S, Tassa A, Qu XP, Garuti R, Liang XH, et al (2005) Bcl-2 antiapoptotic proteins inhibit Beclin 1-dependent autophagy. Cell 122: 927–939. 16179260 10.1016/j.cell.2005.07.002

[16] Bjørkøy G, Lamark T, Johansen T (2006) p62/SQSTM1: a missing link between protein aggregates and the autophagy machinery. Autophagy 2: 138–139. 16874037 10.4161/auto.2.2.2405

[17] Shintani T, Klionsky DJ (2004) Autophagy in health and disease: A double edged sword. Science 306: 990–995. 15528435 10.1126/science.1099993 PMC1705980

[18] Uchiyama Y, Koike M, Shibata M (2008) Autophagic neuron death in neonatal brain ischemia/hypoxia. Autophagy 4: 404–408. 18212531 10.4161/auto.5598

[19] Rami A, Langhagen A, Steiger S (2008) Focal cerebral ischemia induces upregulation of Beclin 1 and autophagy-like cell death. Neurobiol Dis 29: 132–141. 17936001 10.1016/j.nbd.2007.08.005

[20] Wen YD, Sheng R, Zhang LS, Han R, Zhang X, et al (2008) Neuronal injury in rat model of permanent focal cerebral ischemia is associated with activation of autophagic and lysosomal pathways. Autophagy 4: 762–769. 18567942 10.4161/auto.6412

[21] Tian F, Deguchi K, Yamashita T, Ohta Y, Morimoto N, et al (2010) In vivo imaging of autophagy in a mouse stroke model. Autophagy 6: 1107–1114. 20930570 10.4161/auto.6.8.13427

[22] Koike M, Shibata M, Tadakoshi M, Gotoh K, Komatsu M, et al (2008) Inhibition of autophagy prevents hippocampal pyramidal neuron death after hypoxic-ischemic injury. Am J Pathol 172: 454–469. 18187572 10.2353/ajpath.2008.070876 PMC2312361

[23] Carloni S, Buonocore G, Balduini W (2008) Protective role of autophagy in neonatal hypoxia-ischemia induced brain injury. Neurobiol Dis 32: 329–339. 18760364 10.1016/j.nbd.2008.07.022

[24] Wang JY, Xia Q, Chu KT, Pan J, Sun LN, et al (2011) Severe global cerebral ischemia-induced programmed necrosis of hippocampal CA1 neurons in rat is prevented by 3-Methyladenine: a widely used inhibitor of autophagy. J Neuropathol Exp Neurol 70: 314–322. 21412169 10.1097/NEN.0b013e31821352bd

[25] Sheng R, Zhang LS, Han R, Liu XQ, Gao B, et al (2010) Autophagy activation is associated with neuroprotection in a rat model of focal cerebral ischemic preconditioning. Autophagy 6: 482–494. 20400854 10.4161/auto.6.4.11737

[26] Kirino T (2002) Ischemic tolerance. J Cereb Blood Flow Metab 22: 1283–1296. 12439285 10.1097/01.WCB.0000040942.89393.88

[27] Dirnagl U, Simon RP, Hallenbeck JM (2003) Ischemic tolerance and endogenous neuroprotection. Trends Neurosci 26: 248–254. 12744841 10.1016/S0166-2236(03)00071-7

[28] Park HK, Chu K, Jung KH, Lee ST, Bahn JJ, et al (2009) Autophagy is involved in the ischemic preconditioning. Neurosci Lett 451: 16–19. 19103253 10.1016/j.neulet.2008.12.019

[29] Puyal J, Vaslin A, Mottier V, Clarke PGH (2009) Postischemic treatment of neonatal cerebral ischemia should target autophagy. Ann Neurol 66: 378–389. 19551849 10.1002/ana.21714

[30] Zheng YQ, Liu JX, Li XZ, Xu L, Xu YG (2009) RNA interference-mediated downregulation of Beclin1 attenuates cerebral ischemic injury in rats. Acta Pharmacol Sin 30: 919–927. 19574998 10.1038/aps.2009.79 PMC4006642

[31] Schmelzle T, Hall MN (2000) TOR, a central controller of cell growth. Cell 103: 253–262. 11057898 10.1016/s0092-8674(00)00117-3

[32] Sharkey J, Butcher SP (1994) Immunophilins mediate the neuroprotective effects of FK506 in focal cerebral ischaemia. Nature 371: 336–339. 7522303 10.1038/371336a0

[33] Bochelen D, Rudin M, Sauter A (1999) Calcineurin inhibitors FK506 and SDZ ASM 981 alleviate the outcome of focal cerebral ischemic/reperfusion injury. J Pharmacol Exp Ther 288: 653–659. 9918571

[34] Labrande C, Velly L, Canolle B, Guillet B, Masmejean F, et al (2006) Neuroprotective effects of tacrolimus (FK506) in a model of ischemic cortical cell cultures: role of glutamate uptake and FK506 binding protein 12 kDa. Neuroscience 137: 231–239. 16289353 10.1016/j.neuroscience.2005.08.080

[35] Victor ED, Vasyl SN, Lesya VT, Vyacheslav YZ, Alexey AM, et al (2006) Proteasomal proteolysis in anoxia-reoxygenation, preconditioning and postconditioning of isolated cardiomyocytes. Pathophysiol 13: 119–125. 10.1016/j.pathophys.2006.01.003 16597498

[36] Wagner C, Tillack D, Simonis G, Strasser R, Weinbrenner C (2010) Ischemic post-conditioning reduces infarct size of the in vivo rat heart: role of PI3-K, mTOR, GSK-3β, and apoptosis. Mol Cellular Biochem 339: 135–147. 20054613 10.1007/s11010-009-0377-x

[37] Lajoie C, El-Helou V, Proulx C, Clement R, Gosselin H, et al (2009) Infarct size is increased in female post-MI rats treated with rapamycin. Can J Physiol Pharmacol 87: 460–470. 19526041 10.1139/y09-031

[38] Krolikowski JG, Weihrauch D, Bienengraeber M, Kersten JR, Warltier DC, et al (2006) Role of Erk1/2, p70s6K, and eNOS in isoflurane-induced cardioprotection during early reperfusion in vivo. Can J Anaesth 53: 174–182. 16434759 10.1007/BF03021824

[39] Petiot A, Ogier-Denis E, Blommaart EF, Meijer AJ, Codogno P (2000) Distinct classes of phosphatidylinositol 3′-kinases are involved in signaling pathways that control macroautophagy in HT-29 Cells. J Bio Chem 275: 992–998. 10625637 10.1074/jbc.275.2.992

[40] Cui DR, Wang L, Qi AH, Zhou QH, Zhang XL, et al (2012) Propofol prevents autophagic cell death following oxygen and glucose deprivation in PC12 cells and cerebral ischemia-reperfusion injury in rats. PloS One 7: e35324. 22509406 10.1371/journal.pone.0035324 PMC3324553

[41] Zhu C, Wang X, Xu F, Bahr BA, Shibata M, et al (2004) The influence of age on apoptotic and other mechanisms of cell death after cerebral hypoxia-ischemia. Cell Death Differ 12: 162–176. 10.1038/sj.cdd.4401545 15592434

[42] Qin AP, Liu CF, Qin YY, Hong LZ, Xu M, et al (2010) Autophagy was activated in injured astrocytes and mildly decreased cell survival following glucose and oxygen deprivation and focal cerebral ischemia. Autophagy 6: 738–753. 20574158 10.4161/auto.6.6.12573

[43] Vigneron F, Dos Santos P, Lemoine S, Bonnet M, Tariosse L, et al (2011) GSK-3β at the crossroads in the signalling of heart preconditioning: implication of mTOR and Wnt pathways. Cardiovas Res 90: 49–56. 10.1093/cvr/cvr002 21233250

[44] Yuan YJ, Guo QL, Ye Z, Xia PP, Wang N, et al (2011) Ischemic postconditioning protects brain from ischemia/reperfusion injury by attenuating endoplasmic reticulum stress-induced apoptosis through PI3K-Akt pathway. Brain Res 1367: 85–93. 20940001 10.1016/j.brainres.2010.10.017

