# Supplementary figures and images for: Inhibition of Autophagy Contributes to Ischemic Postconditioning-Induced Neuroprotection against Focal Cerebral Ischemia in Rats
Source: PLoS One. 2012 Sep 28;7(9):e46092. doi: 10.1371/journal.pone.0046092 (PMC3461004; doi:10.1371/journal.pone.0046092)

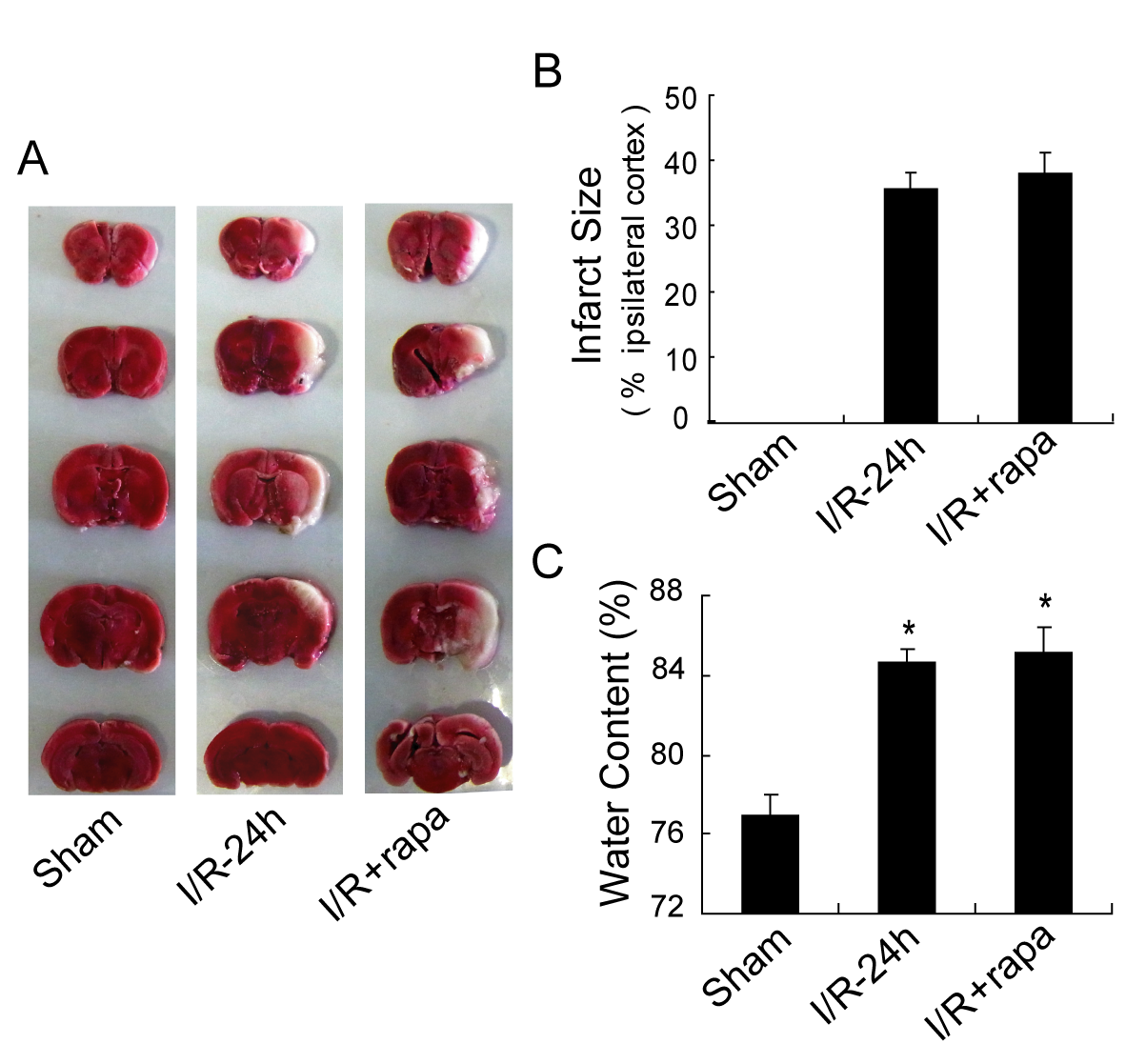

Supplement: Figure S1 — TC staining and brain edema measurement from rat brains in the Sham, I/R-24 h and I/R+rapa groups. Rats were treated with an i.c.v. injection of 35 pmol rapamycin at the onset of reperfusion, and then followed by 24 h reperfusion. (A) Representative infarcts stained with TTC in the Sham, I/R-24 h and I/R+rapa groups 24 h after stroke. (B) Quantification of infarct size from each group at 24 h after ischemia. (C) Quantification of water content from each group at 24 h after ischemia. The results of TTC and water edema measurement showed there was no significant difference between the ischemia-only and rapamycin-treated rats at 24 h after ischemia. n = 8 for each group. *p<0.05 vs. the Sham group. (TIF) [file pone.0046092.s001.tif]
